# Supplementary material for: Novel Friction Law for the Static Friction Force based on Local Precursor Slipping
Source: Sci Rep. 2014 Sep 10;4:6324. doi: 10.1038/srep06324 (PMC4159624; doi:10.1038/srep06324)
Supplement: Supplementary Information — Supplemental Methods [file srep06324-s1.doc]

Novel Friction Law for the Static Friction Force based on Local Precursor Slipping

Yu Katano^1+^, Ken Nakano^2^, Michio Otsuki^3^ & Hiroshi Matsukawa^1^*

^1^Department of Physics and Mathematics, Aoyama Gakuin University, 5-10-1 Fuchinobe, Sagamihara 252-5258, Japan

^2^Faculty of Environment and Information Sciences, Yokohama National University, 79-7 Tokiwadai, Hodogaya, Yokohama 240-8501, Japan

^3^Department of Materials Science, Shimane University, 1060 Nishikawatsu-cho, Matsue 690-8504, Japan

^+^Present address: Applied Technology Engineering Department 2, NetOneSystems Co., Ltd.,

JP TOWER, 2-7-2, Marunouchi, Chiyoda-ku, Tokyo 100-7024, Japan

* Correspondence and requests for materials should be addressed to H.M.

(matsu@phys.aoyama.ac.jp)

Supplemental Methods

A schematic of the apparatus is shown in Supplementary Fig. 1. The slider and base block are transparent rectangles composed of PMMA with a Young’s modulus of 2.5 GPa. The length (*x*), width (*y*), and height (*z*) of the slider are 100, 10, and 20 mm, respectively, and the dimensions of the base block are 120 mm (*x*), 30 mm (*y*), and 40 mm (*z*). The slider is placed on the base block, which is fixed at the center of the apparatus. The bottom edge of this slider has an apex angle of 158°, and the contact area between the slider and the base block is linear in the *x* direction. The linear contact area between the slider and the base block is 100 mm long and 0.8 mm wide. A normal force was applied to the slider via an elastic block composed of silicon rubber with a length, width, and height of 100, 10, and 10 mm, respectively. This elastic block is used to apply a uniform normal force to the top of the slider. Grease is also smeared on the contact surface between the slider and the elastic block to facilitate smooth sliding between the elastic block and the slider. Two load cells separated by 60 mm are used to measure the normal force. The observed values of each load cell are unchanged with 1% of precision, even after bulk sliding of the slider. A shear force is applied to the slider by a leaf spring, with a spring constant *k* = 95 kN/m, via a stainless L-shaped arm, which is fixed to the slider with glue at a height *h* from the top surface of the base block, as shown in Supplementary Fig. 2. The shear force is measured by a strain gauge attached to the leaf spring.

The contact surfaces of the slider and the base block are polished in the *y* direction using sandpaper (#240). The specimens are then washed using detergent, water, and ethanol and are allowed to dry naturally.

The apparatus has a transmissive optical system. The contact area is illuminated by a collimated laser sheet from a green laser diode with a wavelength *λ* = 532 nm. A high-speed camera measures the intensity of the transmissive light. The incident angle of the laser sheet to the contact zone is 60°, which is greater than the critical angle for total reflection at a PMMA-air interface (42°). Therefore, the incident light illuminating the real contact points is transmitted through the interface, and the incident light illuminating the regime that does not contain the real contact points is reflected at the interface. The measured intensity of the transmissive light can be used to quantitatively estimate the local real contact area density. The linear relationship between the increment in the total intensity of the transmissive light and the magnitude of the applied normal load is verified in the absence of a shear force.

The sampling frequency of the data logger is 500 Hz, and all of the measurements are performed in an air-conditioned room with a temperature and relative humidity of approximately 25°C and 30–40%, respectively.

The apparent contact area dependence of the macroscopic static friction coefficient is examined using the slider, from which regions of the lower portion are removed, as shown in Supplementary Fig. 3. The length of the upper surface of the slider is held constant at 100 mm.


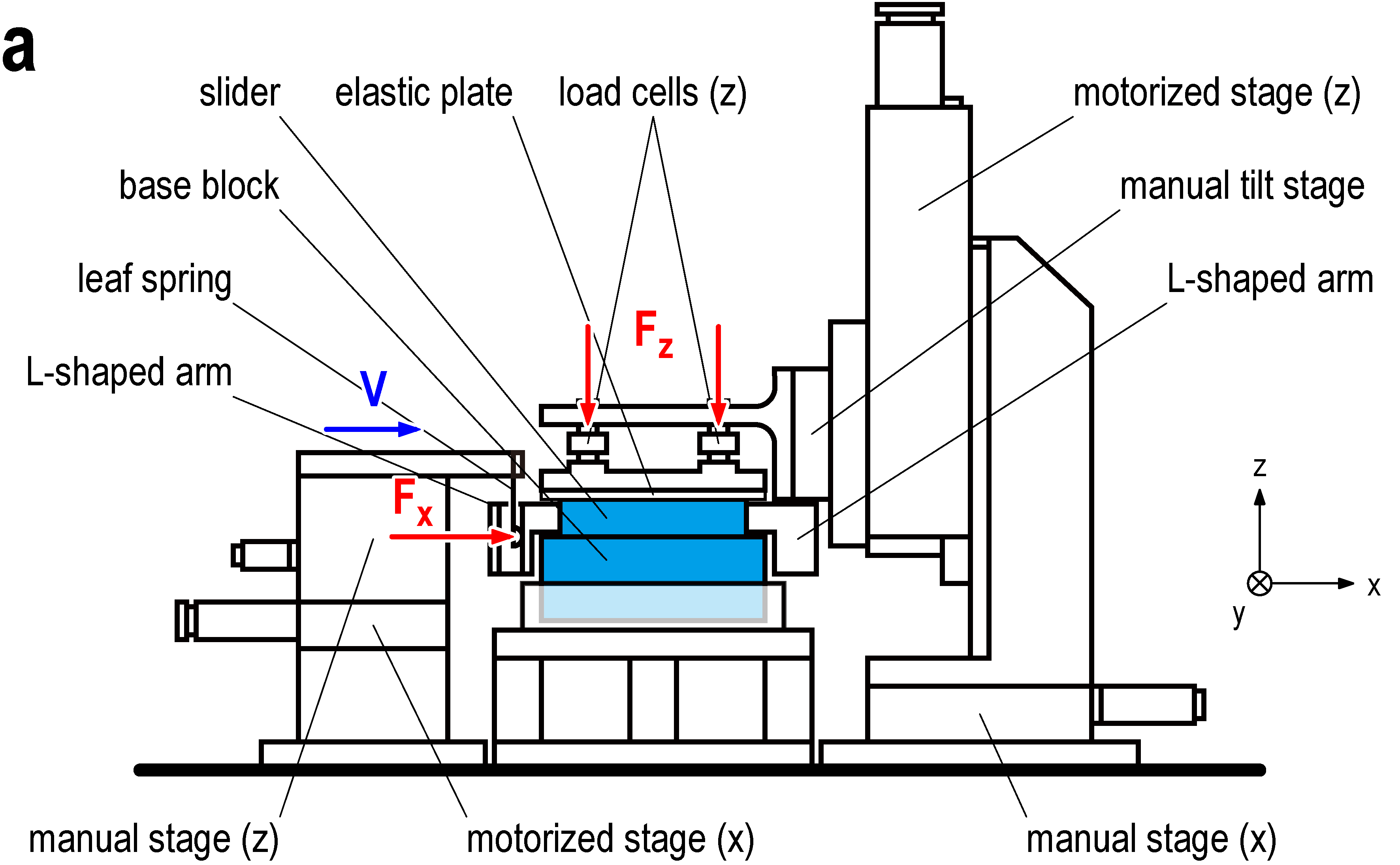


**
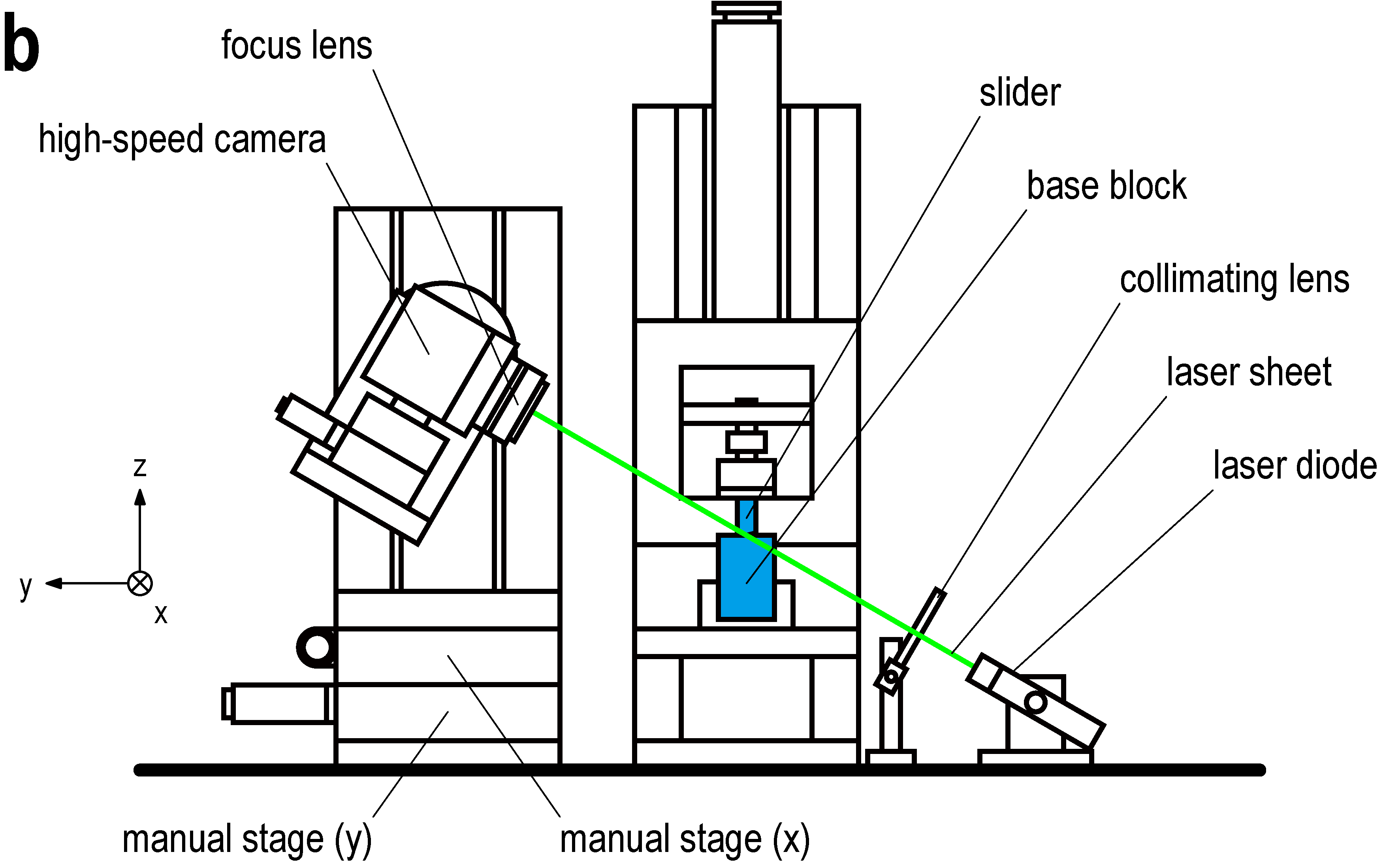
**

**Supplementary Fig. 1: Schematic of the experimental apparatus: (a) front view and (b) side view.**

**
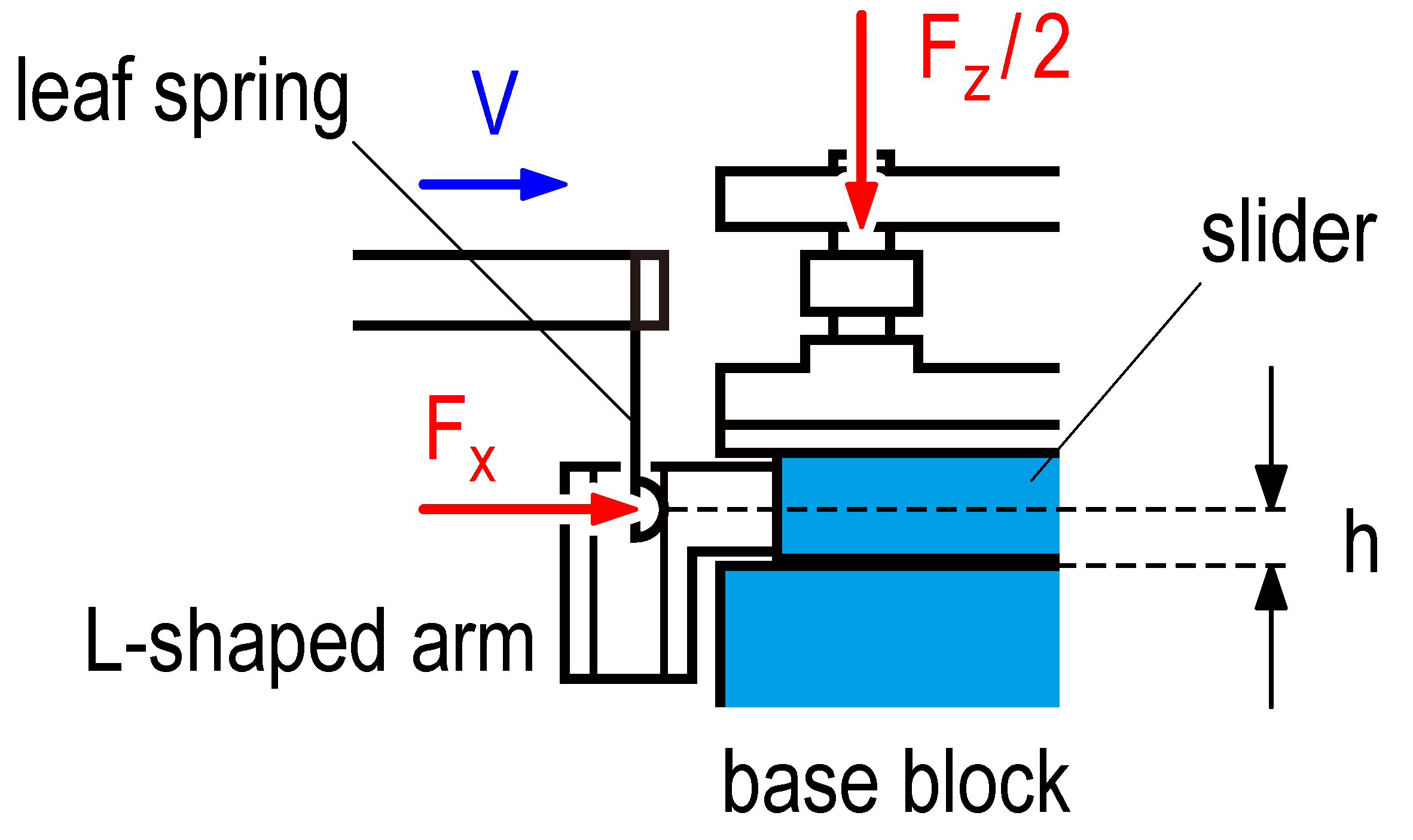
**

**Supplementary Fig. 2: Magnified view of the driving component.**

**
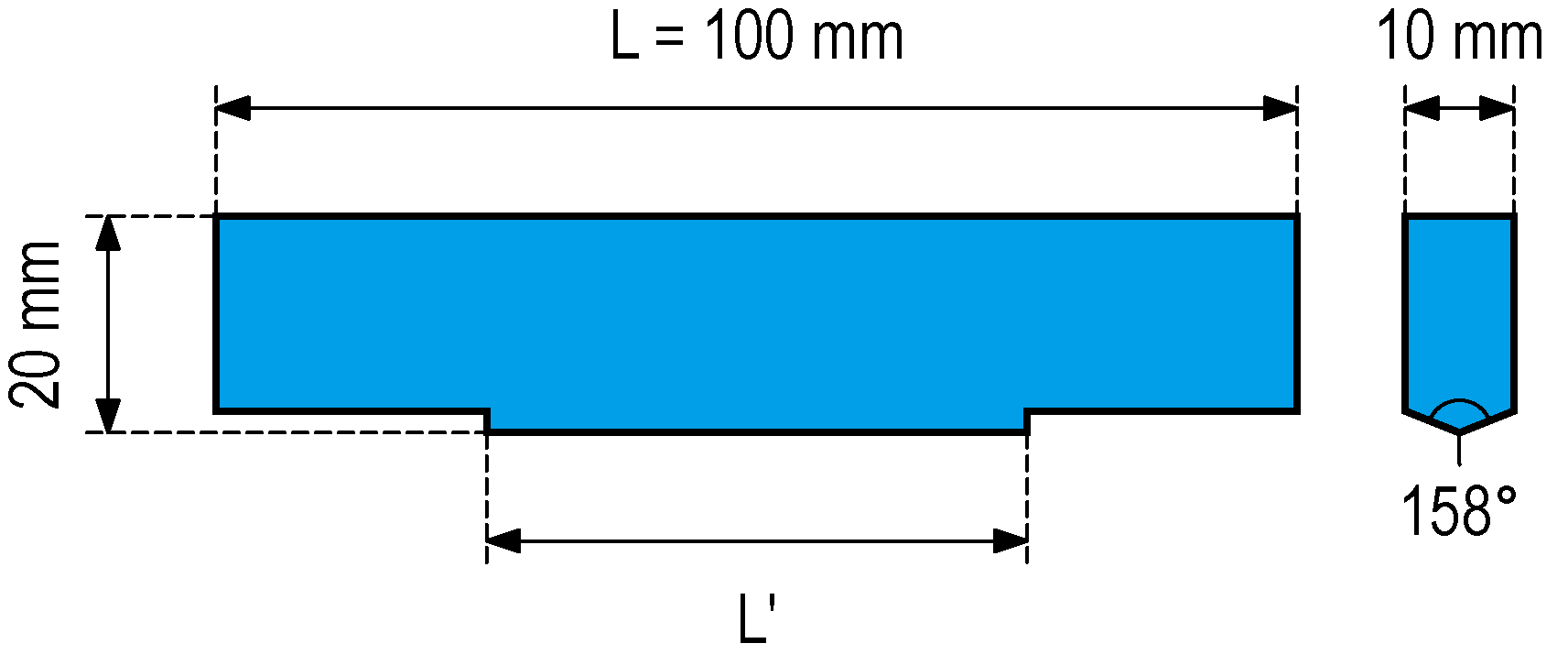
**

**Supplementary Fig. 3: Shape of slider used to investigate the apparent contact area dependence of the static friction coefficient.**
